# Beyond Immersion: A Stage-Based Framework for Cross-Modal Immersive Technologies in Medical Education

**DOI:** 10.7759/cureus.112844

**Published:** 2026-07-17

**Authors:** Christopher M Ahmad, Ambrose Loc T Ngo, Rae-Anne Kastle, Lance Rufino, Shantanu Amin, Brent Evan, Melissa Zolnierz

**Affiliations:** 1 Internal Medicine, Kansas City University of Medicine and Biosciences, Joplin, USA; 2 Osteopathic Medicine, Kansas City University of Medicine and Biosciences, Joplin, USA; 3 Medicine, Kansas City University of Medicine and Biosciences, Joplin, USA; 4 Anatomy, Kansas City University of Medicine and Biosciences, Joplin, USA

**Keywords:** augmented reality, cognitive workload, immersive medical education, learner engagement, medical training analytics, point-of-care ultrasound, presence, simulation-based education, spatial reasoning, virtual reality

## Abstract

Background

Immersive technologies such as virtual reality (VR), augmented reality (AR), and point-of-care ultrasound are increasingly used in medical education, yet most studies evaluate these tools in isolation. As a result, the relationships among immersive experience, cognitive workload, technical performance, and downstream diagnostic assessment remain poorly understood. This exploratory secondary analysis applied the Immersive Learning Engagement-Performance (ILEP) conceptual framework to a staged multimodal dataset to examine whether selected immersive psychological variables were associated with downstream Extension Proficiency Composite and exploratory distal diagnostic performance.

Methods

This theory-informed secondary analysis used participant-level data from a prospective staged protocol involving VR, AR, and ultrasound. All participants completed a VR module before assignment to an AR or ultrasound extension arm, and a subset also completed a dermoscopic image interpretation task as an exploratory downstream diagnostic assessment. Because of the staged design and modest sample size, analyses were conducted using a reduced observed-variable regression approach rather than latent structural equation modeling.

Results

Among participants completing the full final analytic pathway (N = 41), baseline dermoscopy knowledge was the strongest adjusted correlate of dermoscopic diagnostic accuracy (β = 0.86, p < 0.001). Extension Proficiency Composite showed a positive adjusted coefficient that did not reach statistical significance (β = 0.17, p = 0.060), while osteopathic manipulative medicine (OMM) Composite was not independently associated (p = 0.213). In the upstream model, VR-derived psychological presence and cognitive workload were not significantly associated with pooled extension-module performance, and the model demonstrated limited explanatory power (adjusted R^2 = 0.030).

Conclusion

Within this exploratory theory-informed analysis, distal dermoscopic diagnostic performance was primarily associated with baseline knowledge, whereas immersive psychological variables and Extension Proficiency Composite demonstrated limited or non-significant associations.

## Introduction

Immersive technologies are increasingly incorporated into medical education, particularly in learning environments that require spatial reasoning, procedural rehearsal, and active interaction with anatomy. Virtual reality (VR), augmented reality (AR), and point-of-care ultrasound each offer distinct instructional affordances, and prior studies suggest that these modalities can support knowledge acquisition, technical skill development, and learner engagement [1]. However, much of the existing literature remains organized around individual platforms rather than shared educational mechanisms, limiting understanding of how immersive experiences relate to measurable learning and performance outcomes across contexts [2].

Rather than functioning as interchangeable technologies, VR, AR, and point-of-care ultrasound appear to support different forms of learner engagement and skill acquisition within medical education. VR is often associated with immersive conceptual exploration and procedural rehearsal; AR with spatial overlay and anatomical localization; and ultrasound with real-time image acquisition and clinically situated interpretation [3,4]. This distinction is important because educational outcomes may differ not only by modality but also by the cognitive and perceptual demands embedded within each task environment [5].

Performance in immersive environments is shaped not only by exposure itself but also by how learners psychologically and behaviorally engage with the experience. Behavioral engagement, cognitive workload, psychological presence, and learner confidence are frequently measured in simulation-based research, yet they are often treated as parallel descriptors rather than as interrelated components of a broader learning process [6]. As a result, an important gap remains in understanding how immersive engagement translates into knowledge acquisition, technical performance, and downstream learning outcomes within and across immersive modalities.

The present study does not evaluate transfer in the classical sense of direct skill replication across equivalent tasks. Instead, dermoscopy was included as an exploratory downstream diagnostic context requiring visual discrimination and pattern recognition under uncertainty. We selected this task because it shares certain perceptual characteristics with image-based clinical reasoning, allowing examination of whether stage-specific immersive performance demonstrated any observable association with a distinct visual diagnostic task. This should not be interpreted as a formal validation of transfer between educational modalities.

This limitation is further compounded by the tendency to evaluate immersive platforms in isolation. VR, AR, and ultrasound each support different aspects of learning, ranging from immersive exploration to spatial alignment and real-time clinical visualization, but may nonetheless operate through partially overlapping cognitive and engagement pathways [7]. A theory-informed cross-modal conceptual model is therefore useful for examining these relationships across modalities without assuming that the modalities themselves are educationally equivalent. Such an approach shifts the focus from platform-specific outcomes toward the broader mechanisms through which immersive learning may occur.

A theory-informed cross-modal perspective is particularly relevant in immersive education because learners are rarely exposed to these modalities in isolation across real curricula. Instead, students often move between conceptual, spatial, procedural, and interpretive tasks in ways that may engage overlapping but non-identical learning mechanisms [8]. Conceptual models that account for these layered transitions may therefore be more useful than modality-specific evaluations alone when the educational goal is to understand how immersive experiences relate to broader patterns of learning and performance [9].

Within this context, the Immersive Learning Engagement-Performance (ILEP) framework prioritizes psychological engagement variables as the primary upstream anchors of the staged model. While raw technical performance within a novel virtual environment provides a measure of immediate procedural execution, early performance may also reflect differences in familiarity with spatial interfaces, navigation behavior, and virtual interaction demands. By contrast, learner-level psychological variables such as presence and cognitive workload are conceptualized here as proximal indicators of how the immersive instructional environment is cognitively experienced and processed. Modeling these variables as the first stage of the framework therefore allows examination of whether the quality of immersive engagement itself, rather than raw virtual task performance alone, relates to downstream cross-modal proficiency.

The present study applies the ILEP framework as a theory-informed conceptual structure for organizing an exploratory secondary analysis of stage-specific associations within a multimodal immersive medical education dataset. Rather than testing or validating the framework itself, the objective was to examine whether selected learner-level psychological variables, standardized extension-task performance, and a downstream exploratory diagnostic outcome demonstrated interpretable relationships across sequential stages of the parent protocol. Accordingly, this study should be viewed as a hypothesis-generating analysis intended to inform future prospective investigations rather than as a confirmatory evaluation of a unified mechanistic model.

In addition to modality-specific outcomes, an exploratory dermoscopic image interpretation task was included as a downstream diagnostic assessment. This task required fine-detail visual pattern recognition and diagnostic judgment, making it a useful context for examining whether immersive performance signals extended beyond the original training environments [10]. Dermoscopy was therefore not treated as a co-equal immersive modality but rather as a downstream diagnostic task used to explore potential transfer of learning [11].

Dermoscopy provides a useful distal task context because it depends heavily on perceptual discrimination, pattern recognition, and diagnostic decision-making under visual uncertainty rather than on direct procedural replication of the original training activity [12,13]. In that sense, it offers a more demanding test of whether signals derived from immersive educational performance might relate to later interpretive accuracy in a distinct but still image-based clinical domain [11].

This manuscript presents an exploratory integrative analysis across a multi-stage immersive medical education protocol. Although modality-specific outcomes are reported separately in domain-level analyses, the present study intentionally integrates these distinct data streams to examine cross-modal learning patterns that may be less visible when immersive modalities are studied in isolation.

The primary objective of this exploratory secondary analysis was to examine whether selected stage-specific associations within the ILEP conceptual framework were observable in a staged multimodal medical education dataset. Specifically, we evaluated whether psychological presence and cognitive workload during the initial VR experience were associated with standardized extension-task performance and whether extension-task performance demonstrated an adjusted association with an exploratory downstream dermoscopic diagnostic assessment after accounting for baseline knowledge and learner-level covariates.

## Materials and methods

**Figure 1:**
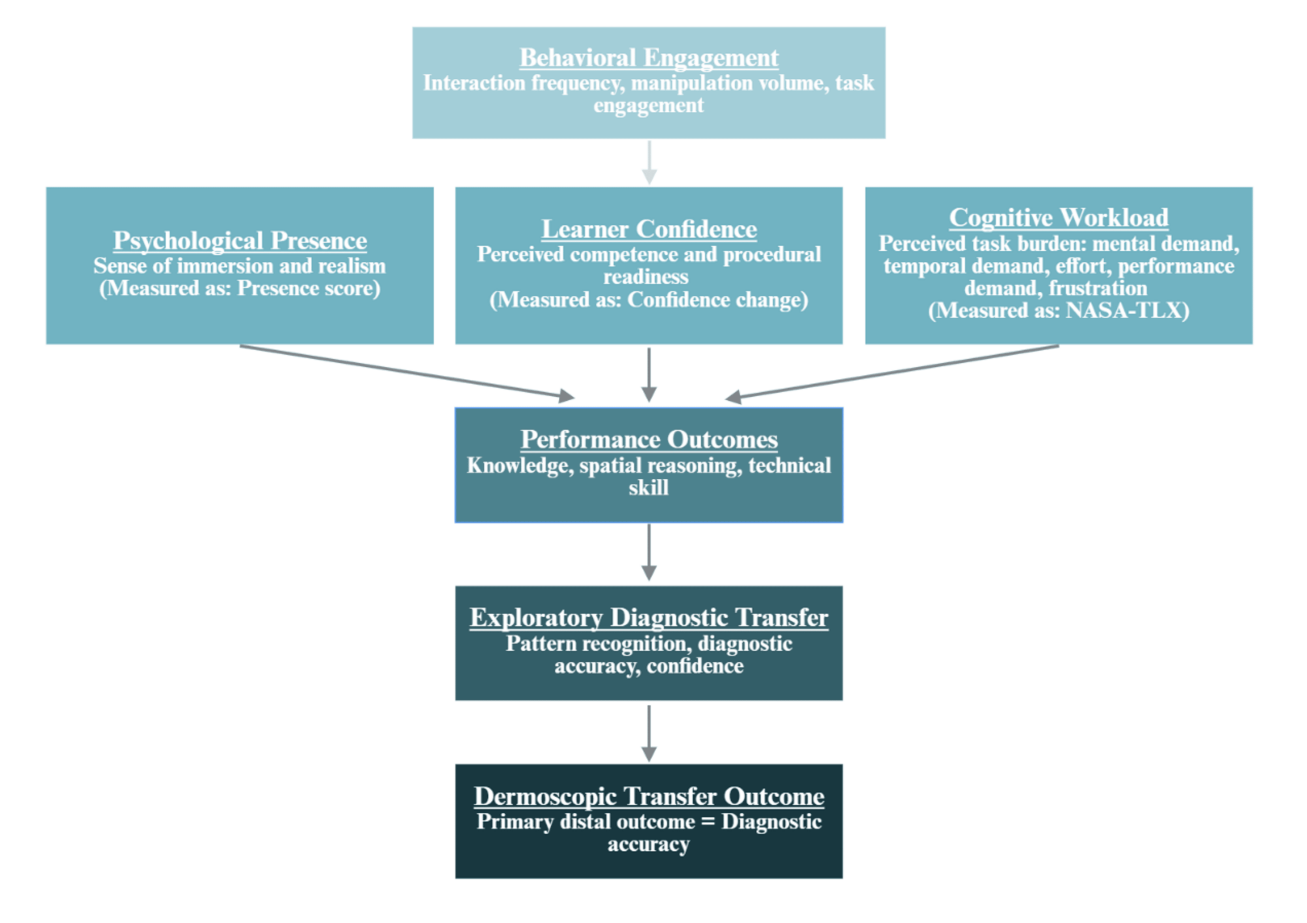
A priori conceptual structure of the Immersive Learning Engagement–Performance (ILEP) framework. The ILEP framework conceptualizes immersive learning as a staged educational process in which behavioral engagement and learner-level psychological factors (including psychological presence, learner confidence, and cognitive workload) may relate to downstream performance across immersive environments, which may in turn relate to exploratory downstream diagnostic performance. This figure represents the full conceptual framework rather than the final fitted analytic model. Because of sample-size and design constraints, only a reduced subset of pathways was examined in the staged observed-variable regression analyses reported in this manuscript. This is the original work of the authors using BioRender. NASA-TLX = National Aeronautics and Space Administration Task Load Index.

The modality-specific educational roles underlying this conceptual framework are summarized in Table 1. Figure 1 illustrates the a priori conceptual organization of the ILEP framework.

**Table 1:** Educational roles and primary outcome domains of the modalities represented within the Immersive Learning Engagement-Performance (ILEP) framework Note: Dermoscopy is presented here as an exploratory downstream diagnostic assessment domain rather than a co-equal immersive instructional modality in the final staged regression model.

Modality/Domain	Educational Role	Primary Outcome Domains
Virtual Reality (VR)	Immersive anatomical exploration and simulated procedural rehearsal within a fully virtual environment	Knowledge acquisition, procedural precision, efficiency, error-adjusted technical performance
Augmented Reality (AR)	Spatial overlay of digital anatomy onto real-world reference frames enabling interactive localization	Spatial reasoning, target localization accuracy, depth perception, plane orientation
Point-of-Care Ultrasound	Real-time anatomical visualization through probe-based image acquisition in a clinically oriented context	Scan accuracy, image quality, acquisition efficiency, real-time anatomical interpretation
Dermoscopy (Exploratory Downstream Diagnostic Domain)	Visual diagnostic task requiring fine-detail pattern recognition and interpretation under uncertainty	Diagnostic accuracy, pattern recognition, confidence, cognitive workload

This study therefore prioritizes relationships across sequential learning stages rather than within-modality improvement, a distinction that remains underexplored in immersive medical education research.

Study design and oversight

This integrative analysis was conducted under approval from the Kansas City University Institutional Review Board, Joplin, USA (IRB #2241234-1) through expedited review procedures. All participants provided written informed consent prior to participation. Immersive sessions were conducted individually in controlled simulation environments to ensure standardized administration across modalities. This study represents a theory-informed secondary analysis of participant-level data derived from a prospective, staged immersive learning protocol conducted under a single multi-arm framework. The parent protocol followed a sequential exposure design in which all participants first completed a core VR instructional session before assignment to either an AR or point-of-care ultrasound extension module. This structure was designed to preserve a shared experiential baseline while allowing examination of both within-modality and cross-modality learning relationships.

The present analysis applied the ILEP conceptual model as a theory-informed analytic lens to examine whether psychological and cognitive features of the initial immersive experience were associated with later cross-modal performance and exploratory distal diagnostic transfer. Because the available sample was modest and the staged design limited simultaneous modeling, the goal was not formal framework validation but exploratory examination of selected hypothesized cross-stage associations. Rather than re-testing modality-specific efficacy outcomes in isolation, this approach focused on identifying structured associations across the sequential immersive learning pathway.

Because the AR and ultrasound extension modules assessed different educational competencies and used different scoring systems, direct comparison of raw performance values would not have been meaningful. Rather than treating these modalities as psychometrically equivalent, we standardized each participant’s primary performance outcome relative to peers within the same extension arm and then carried these standardized values forward as a framework-level indicator of relative learner standing within the assigned extension task. The resulting Extension Proficiency Composite therefore represents relative performance within each participant’s own educational context rather than a common latent construct or direct comparison between AR and ultrasound. This harmonization was used solely to permit exploratory stage-level modeling across the sequential protocol and should not be interpreted as evidence that the two extension modalities measured identical skills or educational domains.

Participants and study flow

A total of 68 medical students were initially recruited for participation in the immersive learning protocol. Of these, 56 participants completed the core VR module and post assessment.

Following completion of the VR session, participants were assigned in approximately equal proportions to either the AR or point-of-care ultrasound extension arm. A total of 55 participants proceeded to an extension module, including 26 participants in the AR arm and 29 participants in the ultrasound arm. One participant did not complete an extension module following VR.

All participants who completed an extension module were eligible for and invited to complete the optional dermoscopic image interpretation assessment as a downstream diagnostic assessment. A total of 41 participants completed this dermoscopy assessment and were included in downstream diagnostic analyses. Attrition across study stages was primarily attributable to scheduling constraints and voluntary non-completion of optional study components.

To preserve comparability across extension pathways, standardization of extension-module performance was performed using the full set of extension completers within each arm prior to restricting analyses to the final analytic subset. Because assignment to the AR or ultrasound arm occurred only after completion of the VR post-assessment, all participants shared a common immersive baseline prior to modality divergence. The staged analytic framework was therefore designed to reflect this dependency structure rather than treat AR and ultrasound as parallel baseline interventions.

Immersive learning protocol

The assessment protocol incorporated structured measurements before and after each immersive exposure. During the VR session, participants completed a baseline survey and pre-intervention knowledge assessment, followed by the immersive instructional module, a post-session survey, and a post-intervention knowledge assessment. The post-VR knowledge score served both as the primary outcome for the VR session and as the cognitive anchor preceding divergence into the extension arms.

Participants in the AR arm completed a pre-session survey, an AR-based spatial localization task, a post-session survey, and a post-intervention knowledge assessment. Participants in the ultrasound arm completed a pre-session survey, a structured ultrasound acquisition task, and a post-session survey, with technical performance evaluated using predefined accuracy-based criteria.

This staged design allowed integration of modality-specific outcomes within a shared sequential framework while preserving a common baseline. Participants contributed data only to those model components corresponding to outcomes available within their assigned extension pathway.

Exploratory downstream dermoscopy assessment

Following completion of the immersive learning sequence, a subset of participants completed a dermoscopic image-interpretation assessment that served as an exploratory downstream diagnostic outcome. Dermoscopy was selected because it requires visual discrimination, pattern recognition, and diagnostic reasoning within a clinically relevant image-based task. It was not intended to function as a direct analogue of either augmented reality or ultrasound, nor as a definitive measure of educational transfer. Rather, it provided an opportunity to explore whether learner characteristics or stage-specific performance demonstrated any observable association with a distinct diagnostic context.

Accordingly, dermoscopy was modeled only as a downstream exploratory diagnostic outcome within the staged analytic framework. Because participants did not receive immersive dermoscopy instruction as part of the parent protocol, performance on this assessment necessarily reflects a combination of baseline knowledge and learner characteristics rather than the effects of immersive training alone. Accordingly, dermoscopy performance should be interpreted primarily as a downstream diagnostic outcome influenced by both prior knowledge and learner characteristics rather than as direct evidence of educational transfer or a direct measure of immersive training effectiveness.

Participants in the final analytic subset completed a dermoscopic image-interpretation assessment designed to evaluate diagnostic accuracy and visual discrimination. Dermoscopic diagnostic accuracy was calculated as the percentage of correctly identified cases across the assessment. Participants also reported task-specific confidence and completed a workload assessment. Baseline dermoscopy knowledge was recorded and retained as a key covariate in downstream diagnostic analyses given its expected influence on diagnostic performance. 

To account for pre-existing domain expertise, a 10-item pre-intervention multiple-choice questionnaire (MCQ) assessing fundamental dermoscopic principles was used as the primary baseline knowledge covariate. This established the learner’s specific starting point for the downstream diagnostic assessment task and allowed the model to isolate the incremental predictive value of immersive extension proficiency.

The selection of dermoscopy as a downstream diagnostic endpoint was grounded in principles of perceptual learning. Unlike procedural tasks that rely more heavily on motor execution, both point-of-care ultrasound and dermoscopy require learners to extract diagnostic patterns from visually complex or “noisy” environments. This allowed exploratory evaluation of whether immersive performance variables were associated with performance in a distinct visually demanding diagnostic task.

Osteopathic manipulative medicine (OMM) variables

The OMM experience was included as an exploratory learner-level variable to account for potential differences in prior tactile and perceptual training. Because all participants had completed at least one year of OMM instruction, these variables were treated as continuous learner characteristics rather than categorical exposure measures.

The OMM Composite incorporated self-reported domains related to palpatory confidence, perceived tactile accuracy, clinical comfort with palpatory diagnosis, and related tactile learning behaviors. OMM variables were not included in the primary extension-performance stage of the ILEP framework, but were examined as exploratory covariates in distal dermoscopy transfer analyses.

ILEP framework and variable structure

The ILEP framework was conceptualized as a staged cross-modal learning process linking immersive psychological and cognitive experience to later performance and downstream diagnostic context. Thus, the fitted analytic model should be understood as a reduced, stage-specific operationalization of the broader ILEP conceptual framework rather than a full structural test of all proposed pathways.

The final fitted framework consisted of two sequential stages. In the first stage, standardized extension-module performance was modeled as a function of two VR-derived psychological variables: psychological presence and cognitive workload. Presence was operationalized using the VR presence total score, and cognitive workload was operationalized using the unweighted mean National Aeronautics and Space Administration Task Load Index (NASA-TLX Mean) score derived from the VR session.

In the second stage, dermoscopic diagnostic accuracy was modeled as an exploratory downstream diagnostic assessment outcome in the subset of participants who completed the dermoscopy assessment. This stage evaluated whether the standardized Extension Proficiency Composite was associated with dermoscopic diagnostic accuracy after accounting for baseline dermoscopy knowledge and OMM tactile proficiency.

Accordingly, confidence-related variables are displayed in descriptive and correlational visualizations for contextual interpretation but should not be interpreted as part of the primary fitted staged regression pathway.

Although broader engagement and telemetry variables were explored during preliminary model development, only variables retained in the final fitted model were included in the primary staged framework in order to preserve parsimony and reduce overparameterization. Learner confidence was included in the broader conceptual ILEP framework as a theoretically relevant affective construct and was explored descriptively and correlationally; however, it was not retained in the final fitted staged regression models. For this reason, confidence is depicted in the conceptual model as a theoretically relevant construct but was not retained in the final fitted regression models and should therefore not be interpreted as part of the primary analytic pathway. 

Data harmonization and variable derivation

To facilitate an integrative analysis across disparate learning environments, all variables were mapped at the participant level using unique identifiers to ensure row-wise integrity and internal consistency.

To support exploratory stage-level analyses across divergent extension pathways, an Extension Proficiency Composite was created by standardizing the primary technical performance outcome within each extension arm (z-scores) before pooling participants into a single analytic variable. Because the augmented reality and ultrasound modules assessed different educational competencies using different scoring systems, this standardization was intended only to represent each participant’s relative standing within their assigned modality. The Extension Proficiency Composite was not designed to represent a common latent construct, psychometric equivalence, or interchangeable technical ability across modalities. Rather, it served as a pragmatic harmonization strategy that enabled exploratory analyses across the staged protocol.

To control for pre-existing expertise within the downstream diagnostic model, baseline dermoscopy knowledge was operationalized using the task-specific pre-intervention dermoscopy MCQ, providing a precise cognitive anchor for the final diagnostic outcome. Standardized extension proficiency values were then carried forward into the final analytical subset (N = 41) for staged modeling.

Sample sizes vary across descriptive summaries according to the stage of the parent protocol from which each variable was derived; however, all staged ILEP regression analyses were restricted to the final analytic subset (N = 41). A broader cross-stage descriptive summary of the immersive, tactile, and downstream diagnostic variables incorporated into the ILEP framework is provided in Table 2.

**Table 2:** Cross-Stage Descriptive Summary Across Parent Cohort Segments. Note: Values are presented descriptively to summarize core variables derived across different stages of the parent protocol. Variables related to dermoscopy and osteopathic manipulative medicine reflect the final downstream diagnostic analytic cohort (N = 41), whereas VR, AR, and ultrasound variables may reflect broader stage-specific parent cohorts. Extension Proficiency Composite reflects standardized within-arm relative performance derived separately from AR and ultrasound extension tasks and is presented as a harmonized cross-modal index for integrative modeling rather than a direct raw comparison between extension modalities. VR: virtual reality; AR: augmented reality; OMM: Osteopathic Manipulative Medicine; NASA-TLX: National Aeronautics and Space Administration Task Load Index

Domain	Variable	N	Mean ± SD
VR Performance	VR Performance Composite	56	0.12 ± 0.87
AR Performance	AR Target Accuracy (%)	26	70.5 ± 12.0
	AR Total Task Time (seconds)	26	405 ± 96
	AR Performance Composite	26	-0.16 ± 0.83
Ultrasound Performance	Ultrasound Performance Composite	29	-0.14 ± 0.67
	Ultrasound Scan Accuracy (%)	29	30.2 ± 23.6
	Ultrasound Image Quality	29	2.28 ± 0.78
	Ultrasound Time to Acquisition (seconds)	29	168 ± 59
Combined Extension Proficiency	Extension Proficiency Composite (AR/Ultrasound)	55	-0.15 ± 0.76
Tactile/OMM Domain	OMM Hours	41	2.9 ± 0.8
	OMM Confidence	41	3.8 ± 0.9
	Palpatory Accuracy	41	3.5 ± 0.9
	OMM Clinical Comfort	41	3.3 ± 0.8
	OMM Composite	41	3.39 ± 0.58
Dermoscopy Domain	Baseline Dermoscopy Knowledge (/10)	41	7.02 ± 1.56
	Baseline Dermoscopy Confidence	41	2.30 ± 0.94
	Dermoscopy Total Correct (/18)	41	11.63 ± 3.05
	Dermoscopic Diagnostic Accuracy (%)	41	64.63 ± 16.93
	Post-dermoscopy Confidence	41	2.17 ± 1.02
	Dermoscopy Confidence Change	41	-0.12 ± 0.78
Cognitive Workload	Dermoscopy NASA-TLX Mean	41	2.67 ± 0.90

Statistical analysis

All analyses were conducted using two-tailed testing with a significance threshold of α = 0.05. Because this exploratory secondary analysis was based on an existing staged dataset, no a priori sample-size calculation was performed for the present regression analyses. Consequently, the study should be interpreted as hypothesis-generating rather than as a definitive test of small or moderate associations. Given the modest sample size and staged dependency structure of the protocol, the ILEP framework was evaluated using a reduced staged observed-variable regression approach rather than latent structural equation modeling. This strategy was selected to maintain interpretability, reduce parameter burden, and avoid overfitting. Accordingly, the present analysis was designed to examine whether selected stage-specific theory-guided associations were consistent with the broader ILEP framework, rather than to estimate a single simultaneous structural model. Given the exploratory nature of this secondary analysis and the staged dependency of the protocol, we utilized a reduced-variable regression approach. This was specifically chosen to maintain a favorable ratio of observations to predictors, ensuring model stability and reducing the risk of overparameterization often associated with smaller sample sizes in complex multimodal datasets.

The first analytic stage modeled the standardized Extension Proficiency Composite among participants who completed the full analytic pathway (N = 41). In this model, VR-derived psychological presence and cognitive workload were entered as predictors of Extension Proficiency Composite.

The second analytic stage modeled dermoscopic diagnostic accuracy within the same analytic subset (N = 41). In this model, standardized Extension Proficiency Composite, baseline dermoscopy knowledge, and OMM composite score were entered as predictors of dermoscopic accuracy. Baseline dermoscopy knowledge was derived from a 10-item pre-intervention assessment, and dermoscopic diagnostic accuracy was calculated from performance on an 18-image interpretation task. Variance inflation factors were also examined to assess multicollinearity among predictors in the Stage II model.

Model fit was assessed using adjusted R², representing the proportion of variance in dermoscopic diagnostic accuracy explained by the predictors included in the Stage II regression model.

Pearson correlation analyses were also performed to characterize relationships among immersive performance measures, dermoscopy readiness variables, workload, confidence, and transfer outcomes. Standardized coefficients (β), unstandardized coefficients (B), standard errors, p-values, and model R² values were reported where applicable. Adjusted R² values were additionally examined to support interpretation of model fit.

No imputation procedures were performed. All primary analyses used complete-case estimation because the staged analytic framework required participant-level data across sequential components of the protocol. Although this approach preserved the internal consistency of the modeled pathways, it reduced the available analytic sample and may have increased susceptibility to selection bias and reduced statistical precision.

Regression assumptions were evaluated through inspection of residual plots and assessment of linearity and homoscedasticity. All modeled relationships were interpreted as associative rather than causal. Statistical significance was interpreted according to the prespecified α level of 0.05. Estimates that did not meet this threshold were reported descriptively to provide complete transparency but were not interpreted as evidence supporting the proposed conceptual relationships. Any discussion of non-significant associations should therefore be understood as hypothesis-generating rather than confirmatory. Because standardized coefficients displayed in summary figures were derived from separate stage-specific models, they are presented for descriptive comparison only and should not be interpreted as coefficients from a single unified equation.

## Results

Participant flow and analytic cohort

A total of 68 medical students were initially recruited for participation in the immersive learning protocol.

Taken together, these findings suggest that baseline dermoscopy knowledge showed the strongest bivariate association with dermoscopic diagnostic accuracy, whereas immersive and tactile variables showed more modest and exploratory relationships.

**Figure 2:**
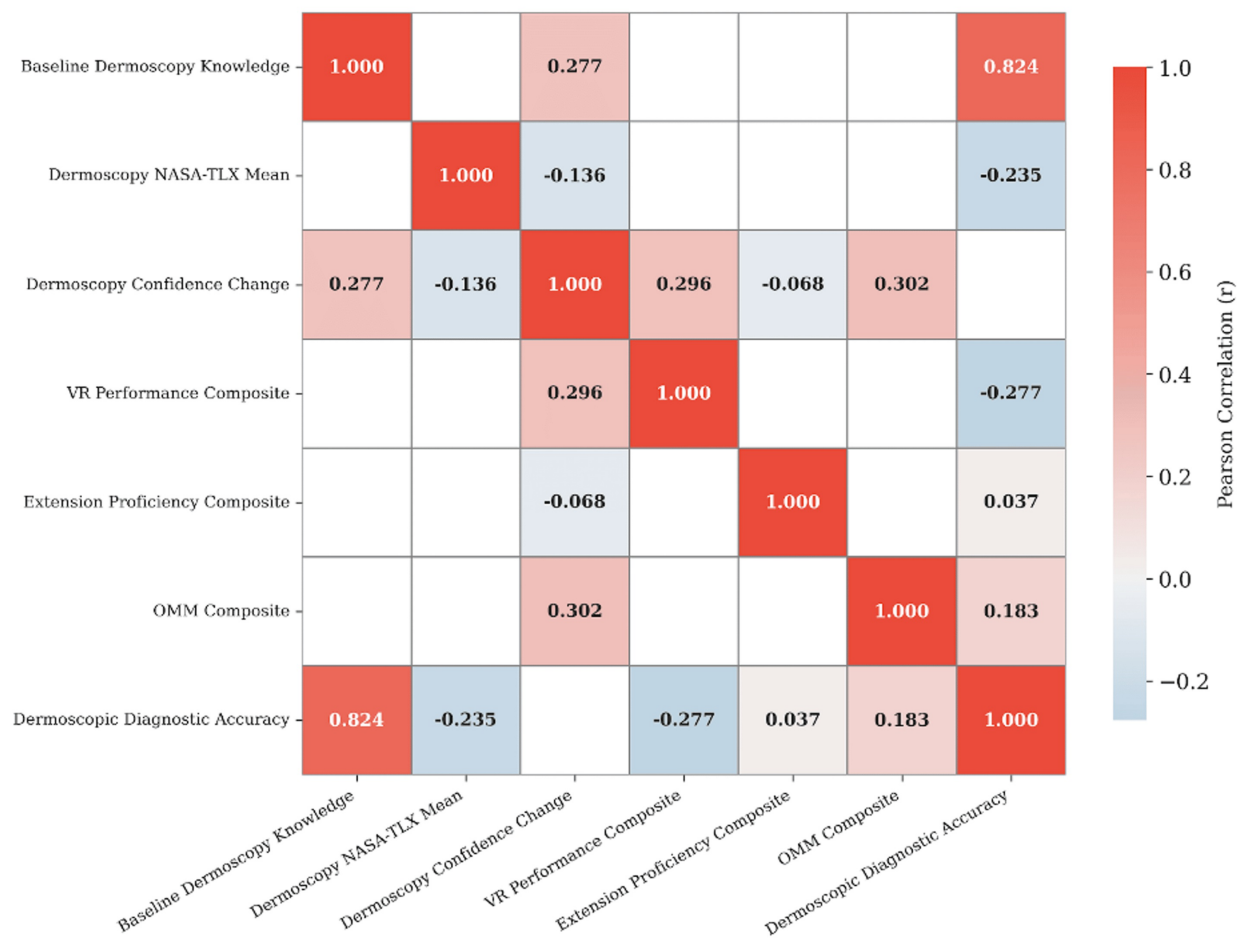
Selected correlation matrix within the fitted ILEP framework. Heat map displaying selected Pearson correlation coefficients among key variables in the final analytic subset (N = 41), including baseline dermoscopy knowledge, dermoscopy NASA-TLX mean, dermoscopy confidence change, VR Performance Composite, Extension Proficiency Composite, OMM Composite, and dermoscopic diagnostic accuracy. Blank cells indicate relationships omitted for visual clarity rather than zero correlation. Warmer colors indicate positive correlations, cooler colors indicate negative correlations, and color intensity reflects correlation magnitude. ILEP: Immersive Learning Engagement–Performance; VR: virtual reality; OMM: osteopathic manipulative medicine; NASA-TLX: National Aeronautics and Space Administration Task Load Index

**Figure 3:**
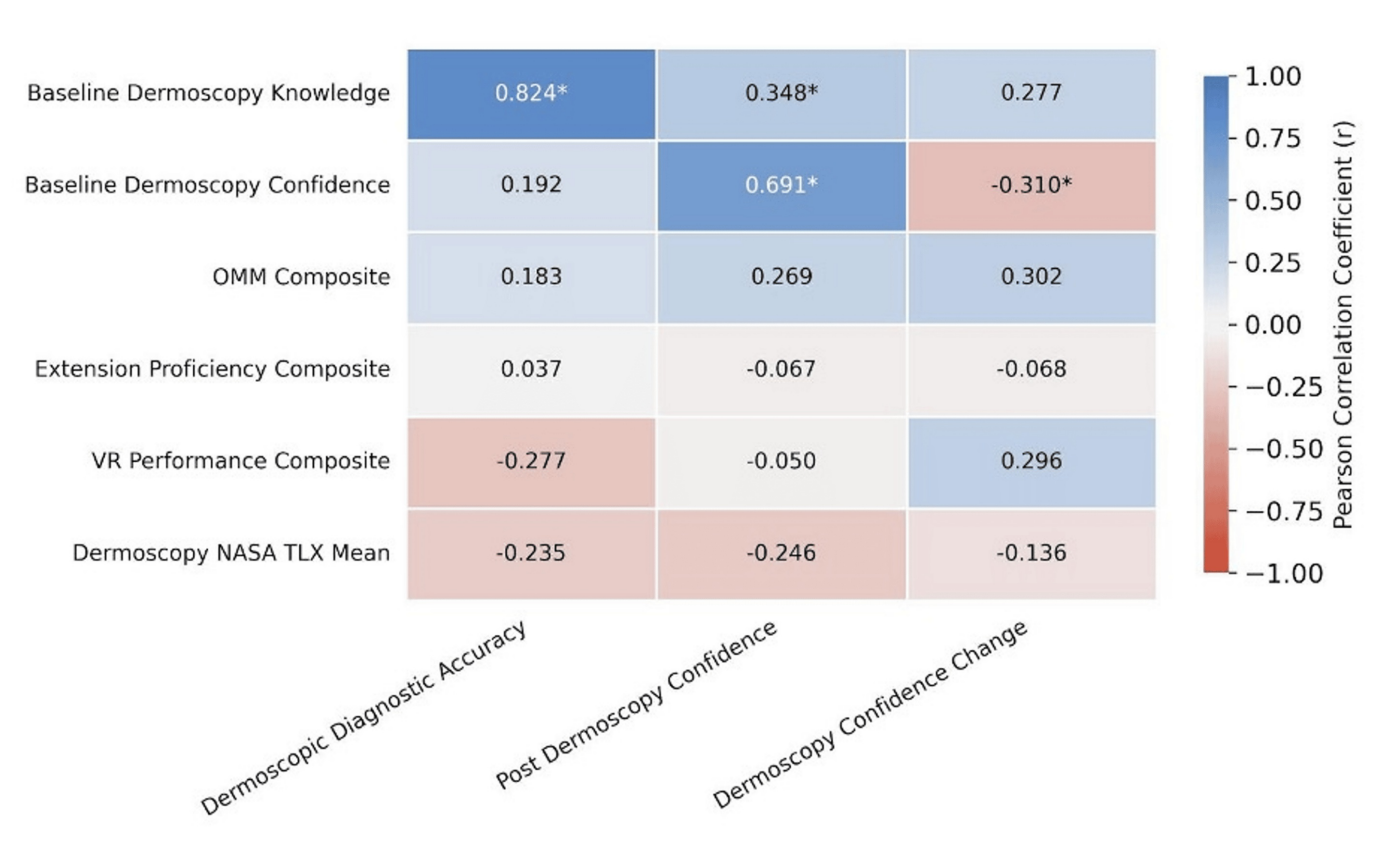
Focused predictor–outcome correlation heat map within the fitted ILEP framework. Heat map displaying Pearson correlation coefficients between selected learner-level predictors and dermoscopy-related outcomes (N = 41). Baseline dermoscopy knowledge demonstrated the strongest association with dermoscopic diagnostic accuracy, whereas immersive performance and Extension Proficiency Composite showed comparatively modest associations. Warmer colors indicate positive correlations, cooler colors indicate negative correlations, and color intensity reflects correlation magnitude. Asterisks indicate p < 0.05. ILEP: Immersive Learning Engagement–Performance; OMM: osteopathic manipulative medicine; VR: virtual reality; NASA-TLX: National Aeronautics and Space Administration Task Load Index

**Figure 4:**
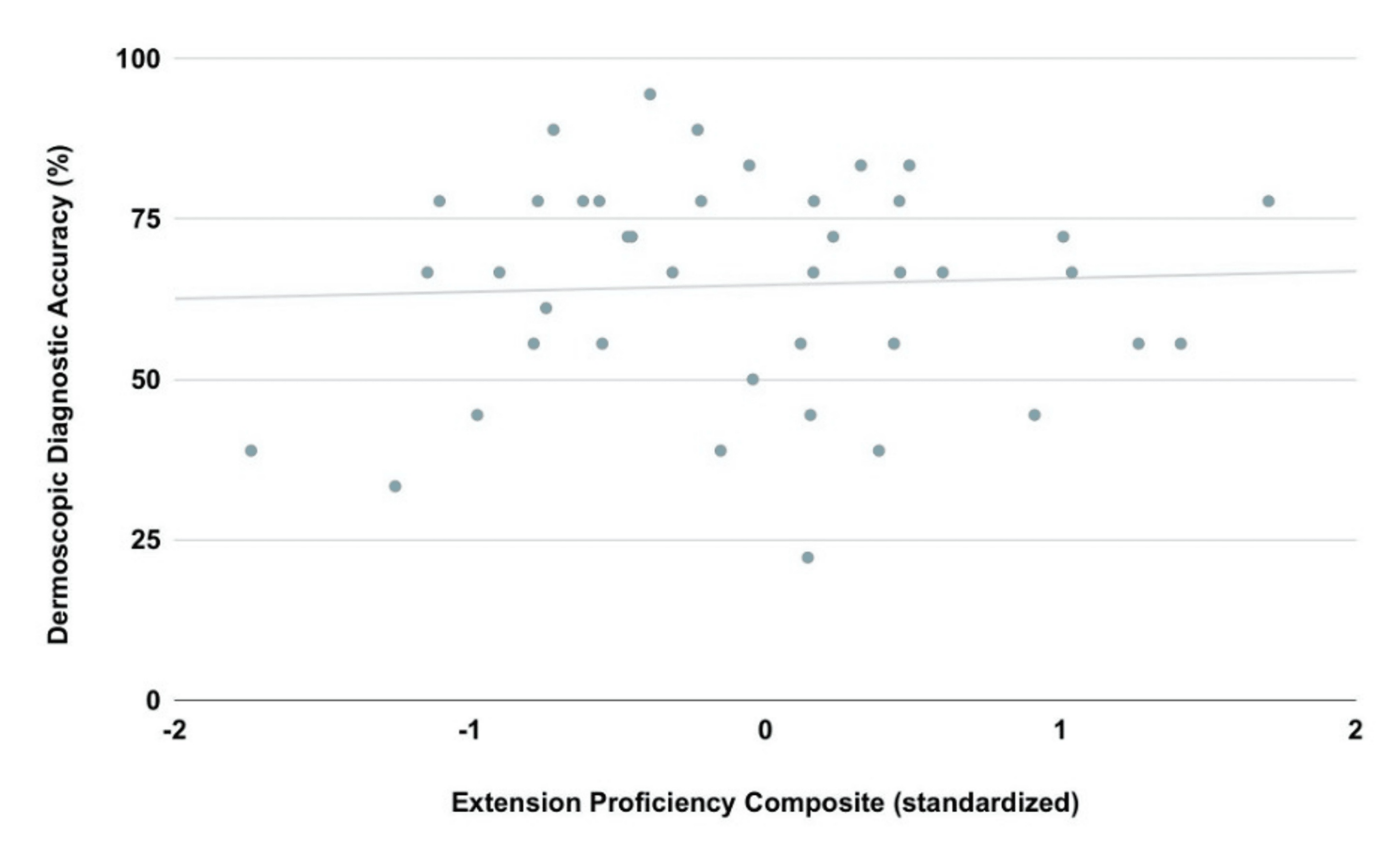
Unadjusted relationship between Extension Proficiency Composite and dermoscopic diagnostic accuracy. Scatterplot showing the bivariate association between pooled standardized Extension Proficiency Composite and dermoscopic diagnostic accuracy among participants completing the final analytic pathway (N = 41). The broad dispersion of points and shallow positive trendline are consistent with a weak zero-order association (r = 0.037, p = 0.820). This unadjusted relationship should be interpreted in the context of the multivariable Stage II model, in which Extension Proficiency Composite demonstrated a positive adjusted coefficient after accounting for baseline dermoscopy knowledge and OMM Composite; however, this association did not reach the prespecified threshold for statistical significance (β = 0.17, p = 0.060). Note: Extension Proficiency Composite = pooled standardized within-arm relative performance index derived from augmented reality and ultrasound extension tasks. OMM: osteopathic manipulative medicine; r: Pearson correlation coefficient.

Figure 2 presents a selective correlation matrix summarizing relationships among key variables included in the fitted ILEP framework. This visualization highlights the overall structure of inter-variable associations, showing that baseline dermoscopy knowledge had the strongest relationship with dermoscopic diagnostic accuracy, whereas immersive performance, extension proficiency, workload, and tactile variables demonstrated weaker and more heterogeneous associations. To facilitate interpretation of outcome-specific relationships, Figure 3 presents a focused heatmap of predictor-outcome correlations for the principal dermoscopy-related transfer measures. Consistent with the primary correlational analyses, baseline dermoscopy knowledge remained the strongest correlate of dermoscopic diagnostic accuracy, whereas immersive performance and pooled extension proficiency demonstrated comparatively modest and largely non-significant associations (Figure 4). Confidence-related outcomes showed a distinct pattern, with baseline dermoscopy confidence demonstrating the strongest association with post-task confidence and an inverse association with confidence change.

Together, these figures reinforce the interpretation that distal diagnostic performance in this cohort was primarily associated with baseline knowledge, with only limited contribution from upstream immersive or cross-modal performance variables.

Stage I analysis: psychological variables associated with extension proficiency

The Stage I regression model evaluated the relationship between VR-derived psychological variables-psychological presence and cognitive workload-and the standardized Extension Proficiency Composite within the final analytic subset (N = 41). The model explained 7.9% of the variance in Extension Proficiency Composite (R² = 0.079). After adjustment for the number of predictors relative to the sample size, the model demonstrated limited explanatory power (R² = 0.079; adjusted R² = 0.030).

VR psychological presence was not significantly associated with Extension Proficiency Composite (B = -0.37, β = -0.45, SE = 0.20, p = 0.082). VR cognitive workload was also not significantly associated with Extension Proficiency Composite (B = 0.32, β = 0.38, SE = 0.21, p = 0.139). Accordingly, the present findings do not provide evidence that either VR-derived psychological presence or cognitive workload independently predicted downstream Extension Proficiency Composite in this cohort.

Stage II analysis: variables associated with dermoscopic diagnostic accuracy

Stage II examined factors associated with the exploratory downstream dermoscopic diagnostic assessment. Consistent with expectations, baseline dermoscopy knowledge emerged as the dominant predictor of diagnostic accuracy, whereas the standardized Extension Proficiency Composite demonstrated only a modest adjusted association that did not meet the prespecified significance threshold. Accordingly, the Stage II findings should be interpreted primarily as describing relationships within this downstream diagnostic context rather than demonstrating cross-modal transfer.

In this model, baseline dermoscopy knowledge demonstrated the strongest adjusted association with dermoscopic diagnostic accuracy (B = 9.32, β = 0.86, SE = 0.96, p < 0.001). Extension Proficiency Composite showed a modest positive adjusted coefficient (B = 3.74, β = 0.17, SE = 1.93), but this association did not meet the prespecified threshold for statistical significance (p = 0.060).

The distal model explained a substantial proportion of the variance in dermoscopic diagnostic accuracy (adjusted R² = 0.700). This model fit was largely attributable to baseline dermoscopy knowledge, whereas the remaining predictors contributed comparatively little after adjustment.

**Figure 5:**
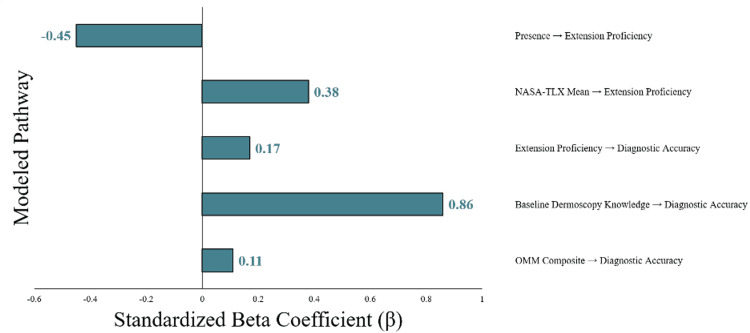
Standardized coefficients from the stage-specific regression models. Stage I shows predictors of standardized extension-task performance, whereas Stage II shows predictors of dermoscopic diagnostic accuracy. Coefficients were estimated from separate regression models and are displayed for descriptive comparison only. Error bars represent standard errors. β: standardized regression coefficient; NASA-TLX: National Aeronautics and Space Administration Task Load Index; OMM: osteopathic manipulative medicine

Overall, these findings indicate that dermoscopic diagnostic accuracy was primarily associated with learners’ baseline task-specific knowledge, whereas Extension Proficiency Composite demonstrated a positive adjusted coefficient that did not meet the prespecified threshold for statistical significance. The unadjusted relationship between Extension Proficiency Composite and dermoscopic diagnostic accuracy is shown in Figure 5. At the bivariate level, the association between these variables was minimal (r = 0.037, p = 0.820). Although the adjusted Stage II model yielded a positive coefficient for Extension Proficiency Composite after accounting for baseline dermoscopy knowledge and OMM Composite, this association did not reach statistical significance (β = 0.17, p = 0.060). Accordingly, the present findings do not provide sufficient evidence to conclude that Extension Proficiency Composite was independently associated with downstream dermoscopic diagnostic accuracy and should be interpreted as exploratory and hypothesis-generating.

Integrated interpretation of the ILEP framework

An integrated summary of the principal modeled relationships is presented in Table 3. For interpretive clarity, the principal directional pathways and their statistical support are summarized in Table 4. Across the staged ILEP framework, baseline knowledge consistently emerged as the dominant determinant of distal visual-diagnostic performance, whereas immersive psychological variables and pooled extension proficiency demonstrated smaller and more exploratory associations.

**Table 3:** Staged observed-variable regression models evaluating the ILEP framework. In Stage I, VR-derived psychological presence and cognitive workload were examined as predictors of pooled extension-module proficiency. In Stage II, Extension Proficiency Composite, baseline dermoscopy knowledge, and OMM Composite were examined as predictors of dermoscopic diagnostic accuracy. Baseline knowledge demonstrated the strongest adjusted association with distal diagnostic performance, whereas Extension Proficiency Composite showed a positive adjusted coefficient that did not reach statistical significance; findings should be interpreted as exploratory and hypothesis-generating. Note: Stage I outcome = pooled standardized Extension Proficiency Composite (model fit: R² = 0.079; adjusted R² = 0.030). Stage II outcome = dermoscopic diagnostic accuracy (% correct). Positive coefficients indicate higher predicted performance with increasing predictor values. Because Stage I and Stage II represent separate fitted models, coefficients should be interpreted within stage rather than compared as if derived from a single simultaneous equation. Multicollinearity was assessed during model evaluation and did not indicate problematic overlap among retained predictors. ILEP: Immersive Learning Engagement–Performance; VR: virtual reality; OMM: osteopathic manipulative medicine; NASA-TLX: National Aeronautics and Space Administration Task Load Index; SE: standard error; B: unstandardized regression coefficient; β: standardized regression coefficient; p: probability value

Stage	Predictor	B (Unstandardized)	SE	β (Standardized)	t	p-value
Stage I: Predictors of Extension Proficiency Composite	Presence	-0.37	0.20	-0.45	-1.80	0.082
NASA-TLX Mean	0.32	0.21	0.38	1.51	0.139	
Stage II: Predictors of Dermoscopic Diagnostic Accuracy	Extension Proficiency Composite (standardized)	3.74	1.93	0.17	1.93	0.060
Baseline Dermoscopy Knowledge	9.32	0.96	0.86	9.64	<0.001	
OMM Composite	3.45	2.75	0.11	1.27	0.213	

**Table 4:** Integrated summary of key modeled relationships within the ILEP framework. The staged model suggests that baseline knowledge was the dominant predictor of distal dermoscopic diagnostic accuracy, whereas immersive psychological and performance variables demonstrated smaller and more exploratory associations. ILEP: Immersive Learning Engagement–Performance; NASA-TLX: National Aeronautics and Space Administration Task Load Index; OMM: osteopathic manipulative medicine; β: standardized regression coefficient; p: probability value; R^2^: coefficient of determination

Relationship	Direction	Statistical Support	Summary Interpretation
Presence → Extension Proficiency	Negative estimated coefficient (not statistically significant)	β = -0.45, p = 0.082	Psychological presence was not significantly associated with the Extension Proficiency Composite.
NASA-TLX Mean → Extension Proficiency	Positive estimated coefficient (not statistically significant)	β = 0.38, p = 0.139	Cognitive workload was not significantly associated with the Extension Proficiency Composite.
Extension Proficiency → Dermoscopic Diagnostic Accuracy	Positive adjusted coefficient (not statistically significant)	β = 0.17, p = 0.060	Positive adjusted coefficient that did not meet the prespecified threshold for statistical significance; findings should be interpreted as exploratory and hypothesis-generating.
Baseline Dermoscopy Knowledge → Dermoscopic Diagnostic Accuracy	Positive	β = 0.86, p < 0.001	Strongest adjusted association with downstream dermoscopic diagnostic accuracy.
OMM Composite → Dermoscopic Diagnostic Accuracy	Positive estimated coefficient (not statistically significant)	β = 0.11, p = 0.213	OMM Composite was not independently associated with dermoscopic diagnostic accuracy after adjustment.
Framework Summary	—	Stage I: R² = 0.079; adjusted R² = 0.030; Stage II: adjusted R² = 0.700	Stage I demonstrated limited explanatory power. Stage II model fit was primarily attributable to baseline dermoscopy knowledge; Extension Proficiency Composite did not demonstrate a statistically significant independent association.

The final fitted framework was broadly consistent with selected components of the ILEP framework as an integrative analytic structure for examining cross-modal learning relationships, while also indicating that not all hypothesized pathways were strongly expressed in this cohort. In particular, no statistically significant association was observed between subjective immersive presence and downstream Extension Proficiency Composite, and cognitive workload likewise was not significantly associated with extension-task performance. These findings suggest that relationships between immersive psychological variables and subsequent technical performance may be more complex and context-dependent than a simple “greater immersion equals greater performance” model would imply. For conceptual comparison, standardized coefficients from the two separate stage-specific regression models are displayed together in Figure 5. These values were estimated from distinct fitted models and should be interpreted within stage rather than as coefficients from a single simultaneous equation.

## Discussion

The staged dataset allowed exploratory examination of participant-level associations across sequential immersive learning activities and a downstream diagnostic assessment. The most consistent finding in this exploratory cross-modal analysis was that distal dermoscopic diagnostic accuracy was primarily driven by baseline task-specific knowledge, whereas immersive psychological variables and cross-modal technical performance contributed comparatively little after adjustment. Although the ILEP conceptual model provided a useful theory-informed structure for organizing these relationships, the observed data were more supportive of stage-specific and outcome-specific associations than of a unified cross-modal mechanism.

A key distinction emerging from the present analysis is that explanatory strength was not uniform across stages of the ILEP framework. The upstream Stage I model linking VR-derived psychological variables to Extension Proficiency Composite demonstrated limited explanatory power (R² = 0.079; adjusted R² = 0.030), indicating that the included psychological variables accounted for relatively little variation in Extension Proficiency Composite. These findings suggest that additional learner-, task-, and modality-specific factors likely contribute to extension-task performance and warrant evaluation in future prospective studies. By contrast, the downstream Stage II model demonstrated substantial explanatory power (adjusted R² = 0.700), largely attributable to baseline dermoscopy knowledge, which emerged as the strongest independent correlate of dermoscopic diagnostic accuracy. Importantly, the high explanatory power of the Stage II model should not be interpreted as evidence that immersive or cross-modal variables broadly drove diagnostic performance; rather, it primarily reflected the dominant contribution of baseline dermoscopy knowledge.

Interpreting the ILEP framework

The present study applied the ILEP conceptual model as a theory-informed organizing structure for exploratory cross-modal analysis. Rather than evaluating VR, AR, and point-of-care ultrasound as isolated instructional modalities, this approach examined whether selected cross-stage associations among immersive psychological experience, downstream technical performance, and distal diagnostic outcomes could be interpreted coherently within a single staged dataset. The results were more supportive of outcome-specific and stage-dependent relationships than of a strongly unified cross-modal mechanism.

The upstream Stage I model demonstrated limited explanatory power (R² = 0.079; adjusted R² = 0.030). These findings indicate that the VR-derived psychological variables included in the present exploratory model accounted for relatively little of the observed variation in the downstream Extension Proficiency Composite. Additional learner-, task-, or modality-specific factors not examined in the current analysis may contribute to variation in downstream extension-task performance and warrant evaluation in future prospective studies.

Within this framework, the most consistent and dominant finding was the central role of baseline dermoscopy knowledge in predicting distal diagnostic accuracy. In the Stage II model, baseline task-specific knowledge demonstrated the strongest independent association with dermoscopic performance and accounted for the majority of explained variance. This suggests that, within the present cohort, pre-existing declarative knowledge remained the strongest correlate of visual diagnostic performance, even when immersive performance variables were considered simultaneously.

The standardized Extension Proficiency Composite demonstrated only a modest, model-dependent association after adjustment. The strong role of baseline knowledge in the present study is consistent with broader educational literature showing that prior declarative understanding often remains a dominant predictor of later task performance, even in environments designed to promote active, experiential, or technology-enhanced learning [14,15]. Immersive tools may enrich learning conditions, but they do not necessarily eliminate the importance of pre-existing conceptual scaffolding when learners are later asked to interpret complex visual or diagnostic information [16].

Taken together, these findings position the ILEP framework not as a single validated mechanistic pathway, but as a structured analytic model for examining how different components of immersive learning contribute variably depending on the educational outcome being measured. Rather than supporting a unified effect of immersion, the present results suggest that immersive learning is better understood as a staged, multidimensional process in which knowledge, performance, and transfer represent related but distinct educational constructs.

This distinction between subjective presence and measurable performance aligns with prior work suggesting that immersive realism, perceptual salience, and learner enthusiasm should not automatically be treated as proxies for educational effectiveness [9,11]. Although presence may enhance motivation, attention, or memorability, its relationship to durable knowledge acquisition and technical execution appears to be conditional rather than uniformly positive across learning contexts [9].

This distinction is consistent with a broader educational literature showing that declarative knowledge, procedural execution, and applied diagnostic reasoning often develop along partially overlapping but non-identical trajectories [17,18]. In simulation-based and technology-enhanced learning environments, improvements in one domain do not necessarily imply proportional gains in the others, particularly when tasks differ in cognitive structure, sensory demands, or performance constraints [19].

Knowledge and performance do not move together

Across the domain-specific analyses, knowledge-based outcomes and technical or spatial performance outcomes did not consistently move together. This dissociation became particularly evident when the ILEP framework was interpreted alongside the modality-specific findings.

Within the VR education domain, participants demonstrated significant immediate improvement in anatomy MCQ performance following the immersive session, but this gain declined at delayed follow-up. In contrast, the VR surgical precision domain showed that technical performance was not meaningfully associated with MCQ gain, reinforcing that procedural execution and declarative knowledge acquisition do not necessarily improve in parallel. Similarly, in the ultrasound domain, baseline knowledge did not independently predict technical scanning performance, and in the AR domain, post-task MCQ performance did not align with spatial task outcomes.

The present exploratory analysis extends this pattern into a downstream diagnostic context. Baseline dermoscopy knowledge remained the dominant predictor of diagnostic accuracy, whereas standardized extension proficiency demonstrated only a modest adjusted association that did not meet the prespecified significance threshold. Accordingly, these findings should not be interpreted as evidence of cross-modal transfer but instead as hypothesis-generating observations for future prospective investigation.

These findings suggest that immersive learning outcomes should be interpreted according to the competency being measured rather than treated as a single educational construct [20]. Educational technologies may differentially influence declarative knowledge, technical execution, and downstream diagnostic reasoning, emphasizing the importance of outcome-specific evaluation when considering curricular integration.

Subjective immersion does not necessarily translate into better performance

Within the present dataset, psychological presence was not significantly associated with the downstream Extension Proficiency Composite. Although the estimated coefficient was negative, the corresponding model explained little variance and was not statistically significant. Consequently, these findings should not be interpreted as evidence that greater subjective immersion impairs subsequent technical performance. Rather, they indicate that no clear association between these variables was demonstrated in this exploratory cohort.

Similarly, cognitive workload was not significantly associated with extension-task performance. Although the estimated coefficient was positive, the available data do not support conclusions regarding beneficial or detrimental effects of workload on later performance. Larger prospective studies will be necessary to determine whether these observed estimates represent reproducible associations or simply sampling variability. This suggests that subjective experience and objective psychomotor acquisition were not closely aligned within this cohort.

Taken together, these findings challenge the common assumption that greater perceived immersion automatically reflects better learning [17]. In some contexts, higher presence may instead reflect novelty, attentional capture, or cognitive absorption that is experientially compelling but not optimally aligned with task demands most relevant to learning [9]. These findings further suggest that perceived realism and learner satisfaction should not be interpreted as direct indicators of educational effectiveness [2].

Cognitive workload was not significantly associated with the Extension Proficiency Composite

VR-derived cognitive workload was not significantly associated with Extension Proficiency Composite in the Stage I model. Because the observed coefficient did not meet the prespecified threshold for statistical significance and the model explained little variance overall (adjusted R² = 0.030), the present data do not support conclusions regarding beneficial or detrimental effects of cognitive workload on downstream extension-task performance.

The broader educational literature suggests that cognitive workload is not inherently detrimental and may, in some contexts, reflect productive challenge rather than overload [21,22]. Moderate levels of perceived task demand have been proposed to accompany effortful processing and strategic problem-solving, whereas excessive or poorly calibrated workload may impair learning and performance [23]. However, the present exploratory analysis did not demonstrate a statistically significant association between VR-derived cognitive workload and downstream Extension Proficiency Composite. Accordingly, these findings neither support nor refute these theoretical perspectives within the current cohort.

Future adequately powered prospective studies are needed to clarify whether relationships between cognitive workload and performance vary according to modality, task demands, and stage of learning.

Exploratory downstream diagnostic associations

One exploratory observation from this study was that Extension Proficiency Composite demonstrated a positive adjusted coefficient for downstream dermoscopic diagnostic accuracy after accounting for baseline dermoscopy knowledge and OMM Composite. However, this association did not meet the prespecified threshold for statistical significance. Because participants did not receive dermoscopy instruction during the immersive protocol and baseline dermoscopy knowledge accounted for most of the explained variance in diagnostic accuracy, these findings should not be interpreted as evidence of educational transfer. Rather, they should be viewed as hypothesis-generating observations that warrant evaluation in larger prospective studies specifically designed to examine relationships between immersive technical performance and downstream diagnostic outcomes.

Overall, baseline task-specific knowledge remained the dominant determinant of downstream dermoscopic diagnostic accuracy, whereas the independent contribution of Extension Proficiency Composite remains uncertain. These findings extend prior immersive education research by examining stage-specific associations with a downstream diagnostic assessment rather than assuming that learning automatically generalizes across educational contexts [11].

Notably, self-reported palpatory and tactile learning characteristics as represented by the OMM composite demonstrated a positive but non-significant association with dermoscopic diagnostic accuracy in the adjusted model. This pattern suggests that OMM-related tactile proficiency may represent a distinct learner competency that neither significantly interferes with nor directly facilitates visual-diagnostic transfer within the present analytic framework.

This is particularly relevant in medical education, where learners are frequently expected to carry perceptual, spatial, and procedural skills from one context into another. The current findings suggest that such transfer may be possible, but that it likely depends on the interaction among prior knowledge, task similarity, and learner-specific performance characteristics rather than on immersion alone.

Not all behavioral metrics reflect productive engagement

A related insight emerging from both the domain-level and integrative analyses is that not all behavioral metrics are pedagogically equivalent. Although immersive technologies generate rich telemetry, the present findings suggest that raw activity volume should not be assumed to reflect meaningful engagement.

The increasing availability of behavioral telemetry in immersive platforms has created new opportunities for educational measurement, but raw interaction volume should not be assumed to reflect meaningful engagement without task-specific interpretation [24,25]. Depending on context, higher behavioral activity may indicate purposeful exploration, corrective behavior, uncertainty, or inefficient trial-and-error rather than deeper learning per se [26].

Within the VR behavioral domain, most telemetry variables, including rotations, zooms, translations, layer toggles, and interaction intensity, did not independently predict knowledge gain or precision outcomes in a robust way. Although interaction intensity was associated with presence, it did not persist as a useful upstream anchor in the final fitted model and was therefore excluded from the fitted ILEP framework. By contrast, in the ultrasound domain, higher behavioral load was associated with poorer technical performance, suggesting that some modality-specific behavioral variables may function less as markers of engagement and more as indicators of inefficiency, correction burden, or novice struggle.

These findings are important because they challenge the increasingly common tendency to treat immersive telemetry as inherently meaningful simply because it is objective and granular [24]. The present work suggests that behavioral signals are only useful insofar as they are interpretable within the demands of the specific task. 

Future immersive education studies should therefore be cautious about collapsing all behavioral metrics into a single construct of “engagement” without regard for context. More informative approaches may involve distinguishing among productive interaction, corrective behavior, novice inefficiency, and purposeful exploration rather than treating all digital activity as educationally equivalent.

Cross-modal heterogeneity

One of the most important implications of this study is that heterogeneity across immersive modalities should not be interpreted as a failure of the framework, but rather as evidence that immersive learning does not operate through a single universal pathway.

The ILEP framework is therefore best understood not as a claim that all immersive modalities operate identically, but as a structure for organizing how different modalities contribute to broader educational development through partially overlapping, non-equivalent mechanisms. This perspective may be more useful for curriculum design than ranking a single “best” modality because it emphasizes aligning technologies with the competencies they are intended to develop.

That distinction is especially important for medical curricula that are increasingly incorporating emerging technologies without always specifying the educational endpoint they are intended to support [2]. The current findings suggest that immersive technologies may be most valuable when selected not because they are novel or highly engaging, but because they are intentionally matched to the competency being targeted.

Educational implications

The present findings are consistent with the possibility that different immersive tools may support different educational competencies, including knowledge acquisition, spatial reasoning, technical execution, and diagnostic interpretation. Accordingly, curricular integration of immersive technologies may be most effective when individual modalities are intentionally aligned with the specific educational competency they are intended to develop [27].

If replicated in larger prospective studies, these findings suggest that immersive technologies may be most effective when aligned with specific educational objectives rather than being viewed as interchangeable instructional platforms [9,27]. Such an approach may allow different immersive modalities to be incorporated according to the competencies they are intended to support, including conceptual understanding, spatial localization, technical execution, and diagnostic interpretation [28].

Second, the repeated dissociation between subjective experience and objective performance suggests that immersive learning evaluations should move beyond satisfaction and perceived realism alone. Although learner enthusiasm and perceived value remain educationally relevant, they should not be assumed to indicate improved competence. The present findings reinforce the importance of incorporating objective performance metrics, retention measures, and transfer-oriented outcomes when evaluating immersive interventions.

Third, because baseline knowledge was the dominant predictor of downstream diagnostic performance in this cohort, immersive technologies should be viewed as complementary educational tools rather than substitutes for foundational knowledge. Future prospective studies are needed to determine how different immersive modalities are best integrated with traditional instruction.

Limitations

Several limitations should be considered when interpreting these findings. First, the final analytic cohort was modest (N = 41), limiting statistical power to detect small or moderate associations and reducing the precision of several regression estimates. Consequently, non-significant findings should not be interpreted as evidence of no association, and the observed relationships should be considered exploratory and hypothesis-generating pending independent replication.

Second, completion of the downstream dermoscopy assessment was optional, resulting in participant attrition and complete-case analysis. Although no significant difference in baseline VR performance was detected between completers and non-completers, unmeasured differences in learner motivation, scheduling availability, or interest in imaging-based tasks may have introduced self-selection bias, thereby limiting generalizability.

Third, the pooled Extension Proficiency Composite required within-arm standardization because augmented reality and point-of-care ultrasound assessed distinct educational competencies. Accordingly, this composite represents relative performance within each participant’s assigned modality rather than a unified psychometric construct or interchangeable technical ability.

Fourth, the dermoscopic image-interpretation assessment served as an exploratory downstream diagnostic outcome rather than a validated measure of educational transfer. Because participants did not receive formal dermoscopy instruction during the study, performance likely reflected baseline knowledge and learner characteristics in addition to any associations with immersive performance.

Fifth, several learner-level variables, including the OMM composite, were based on self-report and may therefore be subject to reporting bias.

Finally, this exploratory secondary analysis was observational and associative. Because the study was conducted at a single institution, the findings should be generalized cautiously to other learner populations and educational settings. Furthermore, causal relationships among immersive experience, technical performance, and downstream diagnostic outcomes cannot be inferred.

Future directions

Future studies should prospectively recruit larger, adequately powered cohorts with mandatory completion of downstream assessments to reduce attrition and improve the precision of estimated associations. Such designs would also permit more sophisticated modeling approaches, including modality-specific analyses and latent-variable methods, while reducing concerns related to selection bias and incomplete follow-up.

Further studies should examine how immersive tools might best be sequenced within curricula rather than evaluated only as isolated interventions. The present findings suggest that different immersive platforms may support different phases of learning, and future work should test whether intentionally sequenced exposure to VR, AR, ultrasound, and other diagnostic modalities can enhance not only modality-specific performance but also retention and transfer over time.

Future work should also place greater emphasis on mechanism discrimination rather than simply documenting whether outcomes improved. Understanding which aspects of immersive experience reflect novelty, cognitive effort, productive struggle, inefficiency, or meaningful skill acquisition will be essential for designing more efficient and educationally sound immersive curricula.

Finally, future studies should examine whether learner-level characteristics, including prior knowledge, perceptual style, spatial ability, confidence calibration, and tactile training, moderate how immersive learning translates into downstream performance. Such work may help clarify which learners benefit most from particular immersive sequencing strategies and under what conditions cross-modal transfer is most likely to emerge. Future prospective multicenter studies with larger cohorts and formal structural equation modeling will be necessary to validate and refine the proposed ILEP framework.

## Conclusions

In this exploratory theory-informed secondary analysis, the ILEP framework served as a conceptual organizing structure for examining stage-specific associations among immersive educational experiences, technical performance, and a downstream diagnostic assessment. Within the present cohort, baseline dermoscopy knowledge demonstrated the strongest association with dermoscopic diagnostic accuracy, whereas immersive psychological and technical variables showed smaller, non-significant, or context-dependent associations. Given the exploratory design, modest sample size, and the absence of statistical significance for several modeled relationships, these findings should be interpreted cautiously. The principal contribution of this study is descriptive and hypothesis-generating rather than confirmatory. Overall, the results indicate that different immersive modalities may relate to distinct educational competencies, including knowledge acquisition, spatial reasoning, technical execution, and diagnostic interpretation, rather than reflecting a single unified educational effect. Within this cohort, relationships between modality-specific performance and the downstream dermoscopic assessment were limited, while baseline task-specific knowledge remained the dominant predictor of diagnostic accuracy. These findings provide an exploratory foundation for future prospective studies designed to evaluate stage-specific relationships across immersive learning environments using larger, adequately powered cohorts and modality-specific analyses.
